# Biological function of resveratrol and its application in animal production: a review

**DOI:** 10.1186/s40104-022-00822-z

**Published:** 2023-02-11

**Authors:** Qingwei Meng, Jiawei Li, Chunsheng Wang, Anshan Shan

**Affiliations:** College of Animal Science and Technology, Northeast Agricultural University, Harbin, 150030 China

**Keywords:** Animal production, Biological function, Health, Pigs, Poultry, Resveratrol, Ruminants

## Abstract

With the prohibition of antibiotics in feed, plant functional substances have been widely studied as feed additives. Resveratrol, a natural stilbene, and a non-flavonoid polyphenol found in plants, possesses antioxidant, anti-inflammatory, and metabolic regulatory features. Resveratrol generated intense scientific and public interest, primarily due to its widely reported ability to prevent cancer, delay aging and alleviate related metabolic diseases. Recently, resveratrol has been studied and applied as a feed additive in animal production. This review focuses on the outline of the absorption and metabolism and biological functions of resveratrol and summarizes the application of dietary resveratrol in animal production up to the present, including pigs, poultry, and ruminants. In pigs, dietary resveratrol improved intestinal health, mitochondrial function, meat quality, and more. In poultry, studies have shown that dietary resveratrol improves growth performance and meat and egg quality and alleviates heat stress induced adverse effects. There are few studies on dietary resveratrol in ruminants; however previous studies have indicated that dietary resveratrol increases nutrient digestibility and reduces methane emissions in sheep. It is hoped that this review could provide a specific theoretical basis and research ideas for the research and application of resveratrol.

## Background

Natural product compounds have recently drawn significant attention from the scientific community for their potent effects on oxidative stress and inflammation. Resveratrol (3,4',5-trihydroxystilbene), a natural stilbene and a nonflavonoid polyphenol, possesses antioxidant [1], anti-inflammatory [2], cardioprotective [3], antimicrobial [4], and anti-cancer qualities [5]. Resveratrol is naturally found in numerous species of plants, including peanuts, grapes, pines, and berries, and assists in responding to pathogen infections. Interestingly, Chinese traditional medicine also contains it in the form of extracts such as those acquired from *Polygonum cuspidatum*.

The research on this chemical started through the “French paradox”, a term generated in 1992, which describes French people with a low incidence of coronary heart diseases despite consuming a diet with high saturated fat [6, 7]. Since then, resveratrol has been broadly studied and demonstrated to have antioxidant, anti-inflammatory, antimicrobial, anticancer, and antiangiogenic effects, with those on oxidative stress possibly being the most important and underlying the others. Accumulating reports have indicated that resveratrol could prevent or slow the progression of numerous diseases, including cardiovascular disease, cancer, and aging, and improve stress resistance ability [8, 9]. The most striking effect of resveratrol is the resistance to cancer and aging. In 1997, resveratrol was shown to have cancer chemo-preventive activity in assays representing three main stages of carcinogenesis, owing to its antioxidant, antimutagen and anti-inflammatory effects [10]. In 2003, Howitz et al. [11] first, demonstrated that resveratrol is an activator of sirtuin deacetylases and could extend the lifespan of *Saccharomyces cerevisiae*. Then in 2006, two critical studies were successively published, and it was found that resveratrol produces changes associated with longer lifespan, including improved insulin sensitivity, peroxisome proliferators-activated receptor gamma coactivator-1 alpha (PGC-1α) activity, mitochondrial function, and motor function [12, 13]. These essential findings further drew the attention of researchers worldwide. Until now, more than 11,000 papers on resveratrol have been indexed by the Web of Science Core Collection.

**Fig. 1:**
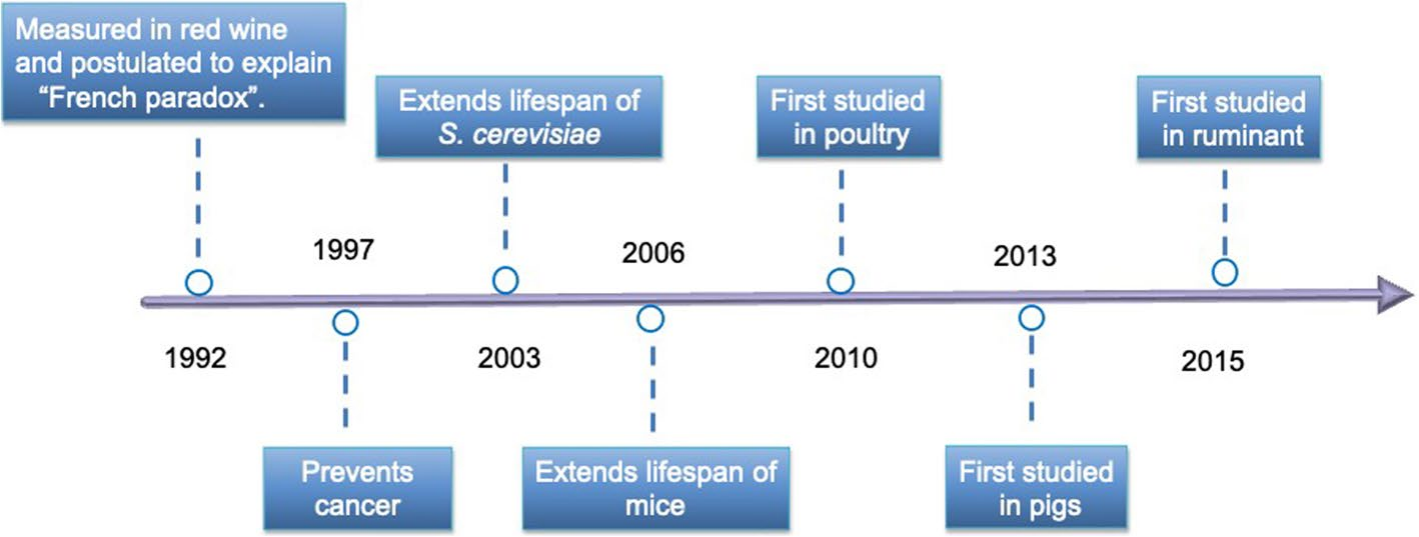
The important research process of resveratrol

Recently, researchers in the animal production area have explored the nutrition regulation effects of dietary resveratrol in animal production (Fig. 1), including pigs [14], poultry [15], and ruminants [16]. Because of the qualities of antioxidant, antimicrobial, anti-inflammatory and metabolic regulation, studies have demonstrated that dietary resveratrol has therapeutic effects on the oxidative stress and inflammation induced by early weaning [17, 18], heat stress [19, 20], mycotoxins [21] and bacterial diseases [14], and beneficial effects on growth development and product quality of animals [22]. This review will focus on the absorption and metabolism and biological functions of resveratrol and summarize the application of dietary resveratrol in animal production up to the present, including pigs, poultry, and ruminants, which may provide essential references for the application and research of resveratrol.

## Structure, source, and absorption of resveratrol

### Structure and source

**Fig. 2:**
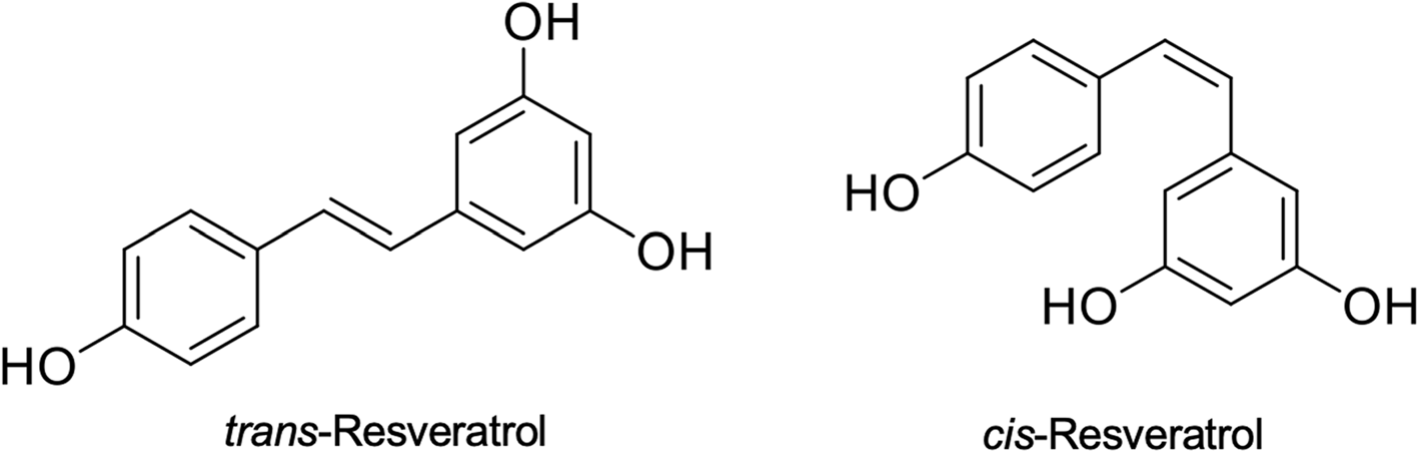
The chemical structure of resveratrol

The structure of resveratrol is stilbene-based and consists of two phenolic rings connected by a styrene double bond to produce 3,4',5-trihydroxystilbene (molecular weight 228.25 g/mol), which occurs in both the *trans-* and *cis*-isoforms (Fig. 2). The chemical structure of resveratrol was characterized in 1940 by Takaoka, who isolated it from the root of the *Veratrum grandiflorum* [23]. Although resveratrol exists in two forms, *trans*-resveratrol is the predominant seen in dietary sources and supplements [23]. As a phytoalexin, resveratrol accumulation in plants is manufactured by a mechanism of resistance to parasites and other adverse conditions, like fungal infection, UV radiation, chemical substances, and generally, stressful factors for the plant [24]. More than 70 species of plants have been discovered to produce resveratrol responding stressful conditions [25]. Additionally, resveratrol is also found in some fruits, such as grapes, blueberries, blackberries, and peanuts. Red wine is the primary source of resveratrol in the Mediterranean diet, which comes from grape skin, seeds, petioles, and woody parts. *Polygonum cuspidatum*, plays a crucial role in Japanese and Chinese traditional medicine and is the richest source of resveratrol [26]. Furthermore, the cultivars of grapes significantly influence the resveratrol content in the skins, leaves and canes of the grapes [27, 28]. For example, Zhang et al. [28] showed that *Vitis vinifera* possessed a high number of *trans*-resveratrol levels than *Vitis labrusca* or the hybrids of *Vitis vinifera* and *Vitis labrusca*, while the grapes used for wine had higher content than that for the table.

### Absorption and metabolism

Despite its well-established beneficial effects, resveratrol has limited bioavailability, and the levels of this molecule found in plasma are very low [29]. The primary reason for low bioavailability of resveratrol is induced by extensive phase-II metabolism after oral administration [29, 30]. In enterocytes and hepatocytes, resveratrol is metabolized to glucuronides and sulfates through UDP-glucuronosyltransferases and sulfotransferases [30]. Resveratrol-3-O-glucuronide, resveratrol-3-O-sulfate, and resveratrol-4'-O-glucuronide were discovered as the most abundant metabolites in humans [31]. Azorín-Ortuño et al. [32] characterized the metabolic profile and pharmacokinetics of resveratrol in the plasma of pigs and identified resveratrol diglucuronide, resveratrol sulfoglucuronide isomers, resveratrol glucuronide isomer, resveratrol-3-O-glucuronide, resveratrol sulfate, and resveratrol, identifying that the most abundant metabolite is resveratrol-3-O-glucuronide. Although the systemic bioavailability of resveratrol is very low, the accumulation of resveratrol in epithelial cells along the aerodigestive tract and potentially active resveratrol metabolites may still have beneficial effects [8, 29]. Additionally, recent studies have shown that the biological effects triggered by resveratrol could be partly explained by modulating gut microbiota composition, especially regarding metabolic syndrome, oxidative stress, and inflammation [33, 34]. Some in vitro and in vivo studies have been performed to clarify the absorption, metabolism, and bioavailability of resveratrol. Intestinal absorption due to a rapid passive diffusion process observed in Caco-2 cells is estimated at 46% in isolated perfused rat small intestines, 77%–80% in vivo in rats, and at least 70% in humans [35]. In humans, resveratrol is rapidly absorbed with the plasma resveratrol concentration peaking approximately 30 min after oral consumption, and about 70%–75% of the absorption is by transepithelial diffusion [4]. Extensive metabolism in the intestine and liver causes oral bioavailability of considerably less than 1%. Henry et al. [36] reported that the uptaking of *trans*-resveratrol is mediated by two transport systems, including passive diffusion and active transport through sodium-dependent glucose transporter 1, and that multidrug resistance protein (MRP)2 seems to be involved in their efflux. Within enterocytes, resveratrol is rapidly metabolized to resveratrol glucuronides and resveratrol sulfates. These metabolites can also effuse back into the small intestine through MRP2, and breast cancer resistance protein 1 and resveratrol conjugates can also efflux through the basal side of the enterocytes through MRP3 [37]. After resveratrol is metabolized by intestinal epithelial cells, it can be transported to the liver and enters hepatocytes through passive and carrier-mediated transport [38]. Resveratrol and its conjugates can be recycled back to the small intestine through the bile or excreted through urine, as reviewed by Nunes et al. [37].

## Biological functions of resveratrol

In this paper, the main biological functions of resveratrol are described, including the antioxidant qualities, anti-inflammatory function, and metabolic regulation function, which have been shown in animals or in vitro models.

### Antioxidant

Oxidative stress is a state of imbalance in which the production of reactive oxygen species (ROS) overwhelms the intrinsic antioxidant capacity defense. ROS are highly reactive and toxic molecules generated from the respiratory chain during mitochondrial oxidative metabolism and the endoplasmic reticulum. Antioxidant system in the body can be divided into enzymatic groups, including glutathione peroxidase (GPX), superoxide dismutase (SOD), catalase (CAT), glutathione reductase, and non-enzymatic groups, including reduced glutathione (GSH), total thiols, ascorbic acid, carotenoids, and α-tocopherol. A plethora of health-beneficial effects of resveratrol occur through its antioxidant qualities. Resveratrol has been indicated to prevent and treat oxidative stress associated with different diseases, including type 2 diabetes [39], hyperglycemia [40], tissue injury [41, 42], Parkinson’s disease [43], neurodegenerative disorders [44], metabolic syndrome [45], and hazardous substance, including ethanol [46], hydrogen peroxide [47], pesticides [48], and mycotoxins [49]. The antioxidant qualities of resveratrol can be explained through its ability to either directly neutralize ROS or indirectly upregulate the expression of cellular defensive pathways and genes. Resveratrol has been shown to be a very effective scavenger of different oxidants, including superoxide anion, hydrogen peroxide, hydroxyl radical, singlet oxygen, nitrogen oxide, and peroxynitrite, associated with the presence of phenolic rings with three hydroxyl groups in positions 3, 4, and 5, and conjugated double bond, and the potential for electron delocalization in the structural molecule as reviewed by Truong et al. [1]. Additionally, extensive research has shown that resveratrol increases the activities of endogenous antioxidant enzymes including CAT, GPX, and SOD, which constitute the primary part of the enzymatic antioxidant defense system against oxidative stress in vitro or in vivo models [46, 50, 51].

As a direct antioxidant agent, resveratrol scavenges diverse ROS/reactive nitrogen species and secondary organic radicals with mechanisms of hydrogen atom transfer and sequential proton loss electron transfer, thereby protecting cellular biomolecules from oxidative damage [1]. Nuclear factor-erythroid 2-related factor 2 (NRF2) is a transcription factor that plays a crucial role in the transcriptional regulation of antioxidant response element-dependent defense genes. In vitro oxidative stress models, previous studies have demonstrated that resveratrol alleviates oxidative stress by activating/regulating the NRF2 signaling pathway in human lung epithelial cells [52], Hep G2 cells [53], PC12 cells [54], endothelial cells [55] and intestinal-epithelial cell [56]. Additionally, in vivo oxidative stress in rats [57] and mice [58], resveratrol was also shown to regulate the NRF2 pathway and prevent the oxidative state. However, resveratrol may behave as an antioxidant or pro-oxidant depending on many parameters, including the dose and microenvironment [59]. Some studies have shown that resveratrol has biphasic concentration-dependent effects, being an antioxidant at low doses and pro-oxidant at high doses [60, 61]. Further studies on the pro-oxidant activity of resveratrol are indicated.

### Anti-inflammatory

The anti-inflammatory qualities of resveratrol have been shown in various animal and in vitro models and contribute to the therapeutic and alleviating effects on disease [8]. Inflammation is a series of cellular and molecular events that help defend the body against infection. Previous studies have reported that resveratrol decreases the production of pro-inflammatory cytokine and inhibits the gene expression associated with inflammation. In a DSS-induced colitis model, dietary resveratrol was shown to reduce the systemic inflammation markers, colonic mucosa prostaglandin E2, cycloxygenase (COX)-2, prostaglandin E synthase and nitric oxide levels in rats [62]. Resveratrol was shown to inhibit the proinflammatory factors interleukin (IL)-1β, tumor necrosis factor alpha (TNF-α), and IL-6 in rats [63, 64]. The significant anti-inflammatory effects of resveratrol have been observed in animal production, especially when responding to oxidative stress [65], mycotoxins [21] or heat stress [66]. Resveratrol shows its anti-inflammatory properties by regulating various pathways. COX is the enzyme in the rate-limiting step of the pathway that manufactures mediators of inflammation. Resveratrol was shown to decrease the prostaglandin E-2 production by inhibiting *COX-2* expression in human fibroblast-like synoviocytes [67]. Additionally, resveratrol could inhibit *COX-2* expression through upstream inhibition of the activity of nuclear factor kappa B (NF-κB) and I-κB kinase [68]. Additionally, anti-inflammatory qualities of resveratrol may also be related to mitogen-activated protein kinases (MAPK) and activator protein-1 pathway as reviewed by Meng et al. [69]. Moreover, resveratrol shows antimicrobial activity against bacteria and fungi, which are undesired in food and plant [70] and are pathogens to animals [4] and humans [71]. The antibacterial activity of resveratrol, impacted by the hydroxy groups at position 4’ [70], is associated with the bacterial species, including Gram-positive bacteria, more sensitive to the resveratrol, whereas the complex structure causes Gram-negative bacteria to show tolerance against it [71]. Previous studies have proved that resveratrol treatment has a therapeutic effect on the inflammation induced by pathogenic bacteria [14, 72]. Therefore, the underlying correlation between resveratrol’s antimicrobial and anti-inflammatory effects should be further investigated.

### Metabolic regulation

**Fig. 3:**
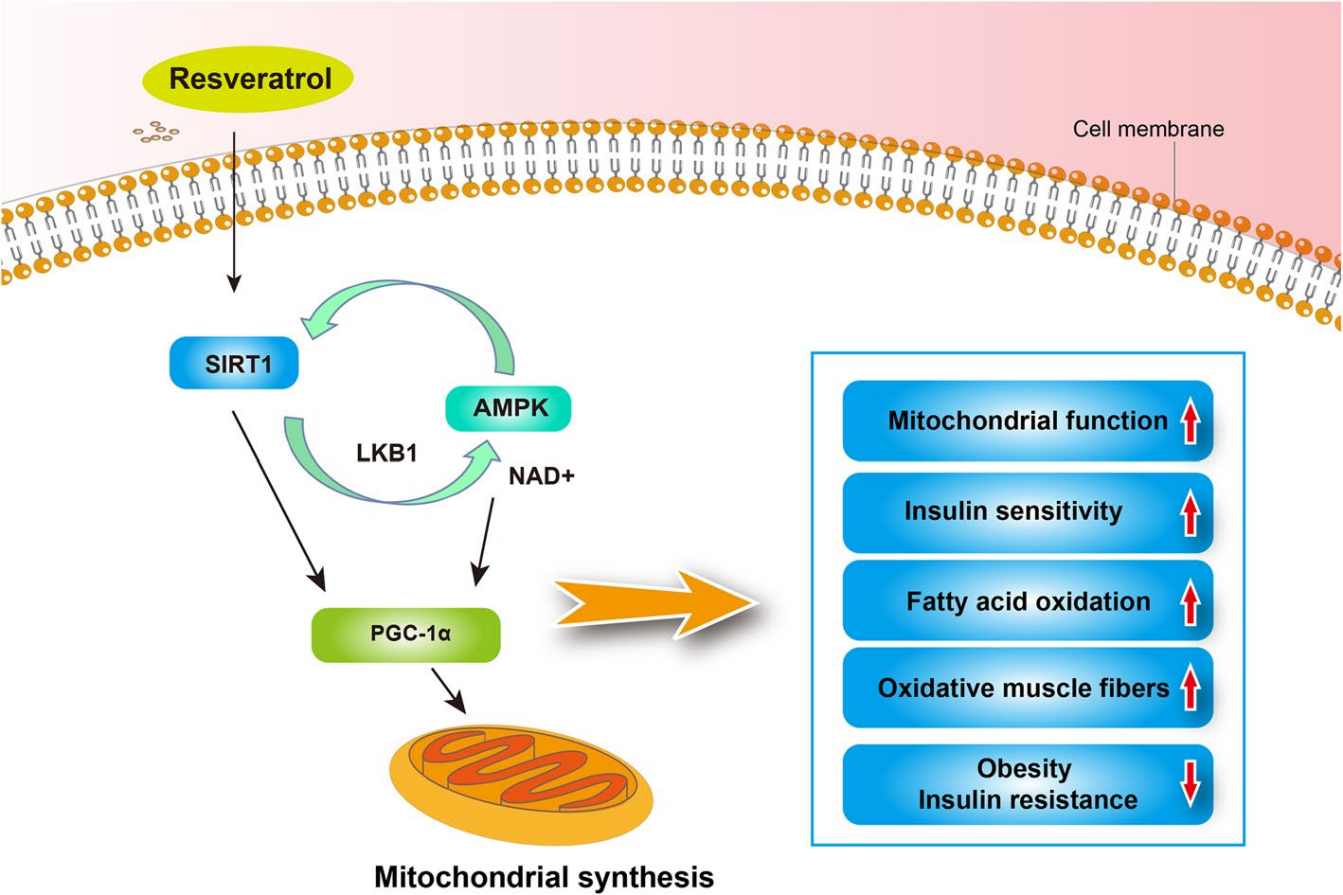
The metabolic regulation effects of resveratrol via SIRT1, AMPK, and PGC-1α [12, 13, 84]. SIRT1, sirtuin 1; AMPK, AMP-activated protein kinase; PGC-1α, peroxisome proliferators-activated receptor gamma coactivator-1 alpha

The metabolic regulation effect of resveratrol has been demonstrated, and it shows beneficial effects on metabolic syndrome and related disorders, including obesity and insulin resistance [73, 74]. The anti-obesity effect of resveratrol has been shown in different animal obesity or high-fat models and humans as indicated by reduced fat deposition and related disorders [75, 76]. In vitro, resveratrol could increase the triacylglycerol breakdown triggered by β-adrenergic activation and impair lipogenesis in human fat cells [77]. In freshly isolated rat adipocytes, resveratrol could decrease basal and insulin-induced lipogenesis from glucose and increase epinephrine-induced lipolysis [78]. Additionally, resveratrol was indicated to influence the viability of adipocytes. Bai et al. [79] reported that resveratrol inhibits pig preadipocyte proliferation and differentiation in vitro by modulation of sirtuin 1 (SIRT1). Rayalam et al. [80] also showed that resveratrol reduces adipogenesis and viability and causes apoptosis in maturing preadipocytes by down-regulating adipocyte-specific transcription factors and enzymes. Resveratrol has been shown to promote fatty acid oxidation. In 3T3-L1 adipocytes and adipocytes obtained from primary mouse embryonic fibroblasts, resveratrol could decrease triacylglycerol content and lipogenic genes, resulting in increased carnitine palmitoyl transferase 1 (a rate-limiting enzyme in fatty acid oxidation), reduced receptor interacting protein 140 (a suppressor of oxidative metabolism), and signs of enhanced flux via the fatty acid beta-oxidation pathway [81]. TNF-α is chronically elevated in the adipose tissues of obese rodents and humans and could induce the production of atherogenic adipokines. Resveratrol exerts a beneficial effect on adipocytes to prevent obesity-induced metabolic alterations through the inhibition of TNF-α-induced changes in atherogenic adipokines in vitro, including plasminogen activator inhibitor-1 and IL-6 [82]. In animal models, resveratrol attenuates insulin resistance and improves insulin sensitivity as well as metabolic complications. Notably, Baur et al. [13] discovered that resveratrol shifts the physiology of middle-aged mice on a high-calorie diet toward that of mice on a standard diet and significantly improves their survival. Resveratrol also produces changes associated with a longer lifespan, including improved insulin sensitivity, decreased insulin-like growth factor-1 levels, increased AMP-activated protein kinase (AMPK) and PGC-1α activity, increased mitochondrial number, and improved motor function [13]. Diminished mitochondrial oxidative phosphorylation and aerobic capacity are associated with decreased longevity [6]. Resveratrol is known to impact mitochondrial function. Lagouge et al. [12] reported that treatment of mice with resveratrol increased their aerobic capacity, as evidenced by their increased running time and consumption of oxygen in muscle fibers, which is because of resveratrol-mediated reduction in PGC-1α acetylation and an increase in PGC-1α activity through activating SIRT1. PGC-1α controls mitochondrial biogenesis and function, which can contribute to fiber-type switching in the muscle. They also found that resveratrol increased fast oxidative fiber percentage in the muscle of mice through regulation of PGC-1α. *SIRT1*, a member of the sirtuin gene family, encodes the most conserved mammalian NAD^+^ -dependent deacetylase enzyme responsible for removing acetyl groups from numerous proteins [83]. Price et al. [84] further showed that mice treated with resveratrol indicated increased mitochondrial biogenesis and function, AMPK activation, and increased NAD^+^ levels in skeletal muscle, whereas *SIRT1* knockouts indicated none of these benefits, and they also found that resveratrol causes a SIRT1-dependent shift toward more oxidative muscle fibers. Calorie restrictions have the potential to extend the lifespan of model organisms and protect against aging-related diseases. In obese humans, resveratrol significantly minimizes sleeping and resting metabolic rates as well as induces metabolic changes, mimicking the effects of calorie restriction [85]. In muscle, resveratrol activates AMPK, increases SIRT1 and PGC-1α protein levels in muscle, and improves mitochondrial muscle respiration on a fatty acid-derived substrate [85]. Thus, resveratrol shows its metabolic regulation primarily though the PGC-1α, SIRT1, and AMPK pathways (Fig. 3).

## Application of resveratrol in swine production

In swine production, the research on dietary resveratrol has mainly been in weaned piglets, finishing pigs, and sows, as shown in Table 1. In this section, we will review the effects of dietary resveratrol based on the growth performance, health, meat quality, and reproductive performance of the pigs.

**Table 1:** Application of resveratrol in swine production

Animal	Dosage	Stress model/ study design	Beneficial effects	Reference
Weaned piglets 21-day-old	300 mg/kg	7-d feeding	Increased villus height in the jejunum; Increased mitochondrial electron transport chain complex I activity, GRP78 and ATF4 protein expression level, and phosphorylation level of IRE1α in the liver	[86, 87]
Weaned piglets 28-day-old	300 mg/kg	28-d feeding	Increased apparent digestibility of crude fat; improved villus height, digestive enzyme activities, antioxidant capacity, and intestinal barrier function; decreased m^6^A enrichment of tight junction protein and antioxidant enzyme-related genes in the jejunum and ileum	[88, 89]
Weaned piglets 28-day-old	150, 300 mg/kg	42-d feeding	Increased IgG level and GPX activity while decreased MDA levels in the serum; enhanced villus and crypt morphology, and mRNA expression of *ZO-1* and *IL-10* in the jejunum	[17]
Weaned piglets 28-day-old	0.1, 0.33, 1.0 g/kg/d resveratrol dry suspension (3% resveratrol)	14-d feeding	Increased peripheral blood lymphocytes, splenic lymphocytes, white cells, neutrophils, lymphocytes, and hemocytes; improved antioxidant enzyme activities, inflammatory response, and antibody levels	[90]
Weaned piglets 28-day-old	300 mg/kg	28-d feeding	Increased IgA and IgG levels in the plasma; increased *IL-10* mRNA expression and *Lactobacillus* copies while decreased *IL-1β* and *TNF-α* mRNA expression in the jejunum and ileum	[91]
Weaned piglets 21-day-old	300 mg/kg	IUGR, 14-d feeding	Increased jejunal villus height; alleviated intestinal oxidative stress by activating NRF2 pathway, inhibited enterocyte apoptosis and decreased intestinal barrier permeability; improved intestinal microbiota composition and increased butyrate production	[92]
Suckling piglets 7-day-old	80 mg/kg BW/d	IUGR, 14-d feeding	Decreased free fatty acid and alanine aminotransferase in serum; increased mRNA expression levels of *LPL*, *GPX1*, *MCP1*, and *TNF-α* while decreased total triglyceride, free fatty acid, protein carbonyl and MDA levels in the liver	[93]
Suckling piglets 4-day-old	600 mg/kg milk	IUGR, 21-d feeding	Increased CAT activity in the serum and liver; increased hepatic ATP production, and *NRF1* mRNA expression	[94]
Suckling piglets 7-day-old	1 g/kg milk	IUGR, 21-d feeding	Increased feed efficiency; increased mitochondrial swelling, complex activities, antioxidant enzyme activities, ATP production, mitochondrial biogenesis, NAD ^+^ and NAD^+^/NADH ratio while decreased AMP/ATP ratio in the liver	[95]
Weaned piglets 21-day-old	300 mg/kg	Diquat challenge, 15-d feeding	Increased SOD activity while decreasing Chiu’s score and cell apoptosis percentage in the jejunum	[41, 96]
Weaned piglets 35-day-old	100 mg/kg	Diquat challenge, 14-d feeding	Increased ADG and ADFI; increased mitochondrial membrane potential, protein expression of PINK1, Parkin, LC3-II, and tight junction protein-related genes while decreased intestinal permeability and ROS production in the jejunum	[97]
Weaned piglets 28-day-old	10, 30, 90 mg/kg	Diquat challenge, 21-d feeding	Improved growth performance; enhanced villus and crypt morphology, antioxidant enzyme activities, and gene expression of antioxidant enzyme in the ileum and jejunum. Increased expression of antioxidant enzyme-related genes and decreased gene expression related to liver inflammation	[65, 98–100]
Weaned piglet 21-day-old	300 mg/kg	DON challenge, 28-d feeding	Improved growth performance; enhanced intestinal morphology, increased goblet cells, and mRNA and protein expression levels of tight junction protein while decreased pro-inflammatory factors levels and mRNA expression in the jejunum; alleviated the negative effects on mRNA and protein expression of mitophagy-related genes in the intestine of piglets challenged with DON	[21, 101]
Weaned piglets 28-day-old	300 mg/kg	DON challenge, 28-d feeding	Increased ADFI; increased villus height and villus height/crypt depth ratio, antioxidant enzyme activities, and mitochondrial membrane potential while decreased MDA and mitochondrial ROS levels in the jejunum	[102]
Weaned piglets 28-day-old	0.2% Respig® (containing resveratrol)	*E. coli* and *Salmonella enterica* challenge, 28-d feeding	Increased ADG and ADFI; increased serum IgG level; decreased fecal *S. typhimurium* and *E. coli* counts	[14]
Weaned piglets 32-day-old	3, 10, 30 mg/kg/d, resveratrol dry suspension	*Rotavirus* challenge, 25-d feeding	Increased CD4^+^ /CD8^+^ ratio; decreased diarrhea index; increased SOD and GPX while decreased MDA, TNF-α and IFN-γ levels in the serum	[103]
Weaned piglets 35-day-old	10, 30, 90 mg/kg BW	*Pseudorabies virus* challenge, 29-d feeding	Increased survival rate, BW, and serum concentrations of IFN-α, IFN-γ, TNF-α, and IL-12; decreased *Pseudorabies virus* copies and lesional scores of brains, lung, kidney, liver, spleen, and heart	[104]
Weaned piglets 28-day-old	150, 300 mg/kg	42-d feeding	Increased expression of slow MyHC, SDH, and MDH activity of and type I fiber proportion; decreased LDH activity and type II fiber proportion	[105]
Growing-finishing pigs, 78.1 kg	300, 600 mg/kg	49-d feeding	Increased pH_24h_, a*, crude protein and myoglobin content and decreased L*, shear force, drip loss in the LM; Reduced backfat depth and visceral adipose tissue weight; Decreased *PPARγ* and *FAS* mRNA levels while increased *HSL*, *ATGL* and *CPT-1* mRNA levels in the adipose tissue; decreased *PPARγ*, *FAS*, *ACC*, and *LPL* mRNA levels while increased *HSL* mRNA levels in the muscle	[22, 106]
Growing-finishing pigs, 65.0 kg	200, 400, 600 mg/kg feed	41-d feeding	Increased *MyHC I* and *MyHC IIa* and decreased *MyHC IIb* mRNA expression; increased SDH and MDH activities in the LM; increased AdipoR1, AdipoR2, AMPK, and PGC-1α expression in LM	[107]
Growing pigs, 24.67 kg	600 mg/kg	119-d feeding	Increased intramuscular fat content and mRNA expression of *PPARγ*, *FAS*, *ACC*, and *LPL* while reduced mRNA expression of *CPT-1*, and *PPARα* in the LM; increased expression of *ssc-miR-181a*, *ssc-miR-370*, and *ssc-miR-21* and reduced expression of *ssc-miR-27a* in the LM	[108]
Newborn piglets 7-day-old	80 mg/kg BW (7–21 days old) 300 mg/kg (21–150 days old)	143-d feeding (NBW vs. IUGR)	Increased GPX activity and *MyHC I* gene expression, reduced protein carbonyl and MDA contents, enhanced fatty acid oxidation via upregulated *PPARα* and targeted genes expression, thereby improving drip loss and b*	[109]
Sows, average parity 4.4	300 mg/kg	Gestation and lactation	Increased weaning weights and ADG 7 d before and after weaning; Increased lactose content in the colostrum and fat content in the milk; alleviated weaning-associated intestinal inflammation and diarrhea and improved intestinal morphology; reduced drip loss and lactic acid level and increased pH_24h_ in the LM of finishing offspring	[18, 110–112]
Sows, average parity 3	300 mg/kg	High temperatures, Gestation and lactation	Increased the number of piglets born alive in both temperature; increased litter weight gain in high temperature; increased antioxidant ability in the plasma and colostrum in both temperature; increased the IgA, IgG, and IgM levels in colostrum under high temperature; Increased *Lactobacillus* and *Alloprevotella* and decreased *Escherichia-shigella* in the faeces of piglets under high temperature	[113]

*SDH* succinate dehydrogenase, *MDH* malate dehydrogenase, *LDH* lactate dehydrogenase, *AdipoR1* adiponectin receptor 1, *PPAR* peroxisome proliferator activated receptor γ, *FAS* fatty acid synthase, *LPL* lipoprotein lipase, *HSL* hormone sensitive lipase, *AMPK* AMP-activated protein kinase, *PGC-1α* peroxisome proliferators-activated receptor gamma coactivator-1 alpha, *ACC* acetyl-CoA carboxylase, *ATGL* adipose triglyceride lipase, *CPT-1* carnitine palmitoyl transferase-1, *CAT* catalase, *GRP78* glucose-regulated protein 78, *ATF4* activating transcription factor 4, *IRE1α* inositol-requiring enzyme 1 alpha, *Ig* immunoglobulin G, *GPX* glutathione peroxidase, *SOD* superoxide dismutase, *MDA* malondialdehyde, *ZO-1* zonula occludes 1, *IL* interleukin-10, *TNF-α* tumor necrosis factor α, *NRF2* nuclear factor erythroid 2-related factor 2, *GSH* glutathione, *MCP1* monocyte chemotactic protein 1, *NAD* + nicotinamide adenine dinucleotide, *NADH* reduced form of nicotinamide adenine dinucleotide, *PINK1* PTEN-induced putative kinase 1, *LC3-II* microtubule-associated protein light chain 3-II, *DON* deoxynivalenol, *ROS* reactive oxygen species, *IFN* interferon-α, *ADG* average daily gain, *ADFI* average daily feed intake, *BW* body weight, *NRF1* nuclear respiratory factor 1, *MyHC* myosin heavy chain, *LM* longissimus muscle, b* yellowness, a* redness, L* lightness

### Growth performance

Under normal physiological conditions, most previous studies failed to observe the improved effects of dietary resveratrol on the growth performance of pigs. However, dietary resveratrol benefits the growth performance of pigs under special physiological conditions, stress, and infection status. Intrauterine growth retardation (IUGR) is usually ascribed to “uteroplacental insufficiency”, a common pregnancy complication that induces low birth weight and retards the growth development of piglets after birth. IUGR newborn piglets ingested 1.0 g resveratrol per kg of milk dry matter from 7 to 21 days of age showed improved average daily milk dry matter intake and feed efficiency. Still, dietary resveratrol failed to improve the average daily gain (ADG) of IUGR piglets [95]. Additionally, dietary resveratrol alleviates the adverse effects of oxidative stress, mycotoxin, or pathogens on growth performance in weaned piglets. In diquat-induced oxidative stress models, the supplementation of resveratrol alleviated harmful effects caused by oxidative stress, increasing the ADG and average daily feed intake (ADFI) and reduced feed to gain ratio (F/G) of weaned piglets [65, 97]. Deoxynivalenol (DON), a significant mycotoxin found in food crops and livestock feed, usually induces oxidative stress, inflammation, and growth inhibition. In a recent study, Qiu et al. [21] found that dietary resveratrol increased the final body weight (BW) and ADG, decreased F/G and tended to increase the ADFI of weaned piglets fed normal and DON diets (3.8 mg DON/kg diet). However, the study by Hong et al. [102] failed to observe ADG and F/G of piglets after the DON challenge but found that dietary resveratrol increased the ADFI of DON-challenged piglets. For pathogens, supplementation of additive based on resveratrol increased ADG and ADFI in a piglet model infected with *E. coli* and *Salmonella enterica*, while the increased survival rate and BW were observed when the resveratrol was supplemented at 10–90 mg/kg BW in a weaned piglet model infected with *Pseudorabies virus* [14, 104]. These studies reported that dietary resveratrol supplementation is conducive for piglets to resist adverse stress factors and infection status and plays a beneficial role in growth performance.

### Meat quality and fat deposition

In pigs, previous studies have discovered that dietary resveratrol showed significant improvement in meat quality. Zhang et al. [22] reported that dietary resveratrol increased the pH_24h_ and meat color redness (a*) and reduced the meat color lightness (L*), shear force, and drip loss of *longissimus* muscle. IUGR, which always causes to abnormal growth and metabolism and reduces meat quality [114]. In a recent study, Cheng et al. [109] discovered that dietary resveratrol supplementation in IUGR pigs from the sucking period to the marked age reduced the drip loss at 24 h and tended to reduce the drip loss at 48 h and yellowness (b*) in the *longissimus* muscle. Meat purchasing decisions are influenced by color more than any other quality factor because consumers use discoloration as an indicator of freshness and wholesomeness [115]. Usually, meat with a high drip loss percentage has an unattractive appearance and therefore has low consumer acceptance, which leads to loss of sales [116]. Thus, the beneficial effects of resveratrol on the meat quality have certain economic value. The meat quality of animals is closely related to muscle fiber type and oxidative stress state. The increased effects of resveratrol on the meat quality of pigs are majorly due to its antioxidant property and muscle fiber-regulating abilities. The antioxidant function of dietary resveratrol in muscle has been shown, as indicated by the improved antioxidant enzyme activities, and related gene expression and reduced oxidative stress markers, including malondialdehyde (MDA) and protein carbonyl [22, 109]. Notably, the muscle fiber-regulating qualities of dietary resveratrol were found in pigs. Muscle fiber type, which is often defined using the isoforms of the myosin heavy chain (MyHC), was closely related to postmortem metabolic rate and meat quality traits. According to the main MyHC isoforms found in adult mammalian skeletal muscles, four single MyHC isoforms have been identified in ATPase-based fiber types: MyHC I in slow-oxidative type I, MyHC IIa in fast oxido-glycolytic type IIa, MyHC IIb in fast glycolytic type IIb and MyHC IIx in fast glycolytic type IIx [117, 118]. Previous studies have demonstrated that higher proportion of IIb fibers causes poor quality of pork and is related to higher L* and lower water-holding capacity and increased the rate and extent of postmortem pH decline [119, 120]. In weaned piglets, a recent study discovered that dietary resveratrol supplementation increased the expression of slow MyHC and the proportion of type I fiber, and reduced the proportion of type II fiber, proposing that resveratrol promotes muscle fiber-type transformation from type II to type I in piglets [105]. In growing-finishing pigs, dietary resveratrol also increases the mRNA expression of *MyHC IIa* and reduces the mRNA expression of *MyHC IIb*, with reduced myofiber cross-sectional area of the *longissimus* muscle. Our previous studies found that maternal dietary resveratrol supplementation increased the mRNA and protein expression of MyHC I and reduced the mRNA and protein expression of MyHC IIb in the *longissimus* muscle of finishing pigs. The drip loss and lactic acid level were reduced, and the pH_24h_ of *longissimus* muscle was increased by maternal dietary resveratrol supplementation [111]. Type IIb fibers have a more glycolytic potential than other types of fibers. Additionally, previous studies have shown that dietary resveratrol in rats increased the ratio of oxidative to glycolytic type muscle fibers and reduced MyHC IIb expression [12]. The reduced lactate content and glycolytic potential in the *longissimus* muscle of pigs and reduced lactate dehydrogenase activity in mouse C2C12 myotubes induced by dietary resveratrol were also observed [22]. The mechanism of muscle fiber-regulating qualities of dietary resveratrol may be because of the regulation of the mitochondrial function and AMPK/SIRT1/PGC-1α pathway [12, 105, 121].

In pigs, resveratrol supplementation in the diets of growing-finishing pigs were shown to decrease the backfat depth at the first rib and the last lumbar vertebra and average backfat depth [22]. Similarly, Zhang et al. [106] reported that resveratrol supplementation reduced the levels of triacylglycerol, total cholesterol, and leptin in serum and the weight of visceral adipose tissue of pigs. Additionally, they also found that the mRNA expression of peroxisome proliferator activated receptor (*PPAR*)γ and fatty acid synthase (*FAS*), and the activities of FAS and lipoprotein lipase (LPL) were reduced by resveratrol, while the mRNA expression of hormone-sensitive lipase (*HSL*), adipose triglyceride lipase (*ATGL*) and carnitine palmitoyl transferase-1 (*CPT-1*), and activities of HSL and CPT-1 were increased by resveratrol [106]. In vitro, resveratrol was found to reduce lipid accumulation and no-esterified fatty acid release in porcine preadipocytes, with increased *SIRT1* expression [122]. ATGL is an essential triglyceride hydrolase that promotes the catabolism of stored fat in adipose tissues, which catalyzes the initial step in triglyceride hydrolysis in adipocyte lipids.In vitro exposure of cultured adipocytes to resveratrol for 24 h increased the mRNA levels of *ATGL* and reduced the lipid accumulation [123]. Interestingly, long-term dietary resveratrol supplementation (119-d feeding) increased intramuscular fat content in pigs; moreover, dietary resveratrol upregulated the mRNA expression of *PPARγ*, *FAS*, acetyl-CoA carboxylase (*ACC*), and *LPL* and downregulated the mRNA expression of *CPT-1*, *SIRT1*, and *PPARα* in *longissimus* muscle, which may be related to enhanced expression of *ssc-miR-181a*, *ssc-miR-370*, and *ssc-miR-21* and reduced expression of *ssc-miR-27a* [108]. Similarly, another study performed by Cheng et al. [109] explored the long-term influence of dietary resveratrol from the sucking period (7 d) to slaughter (150 d) on normal birth weight (NBW), and IUGR pigs observed a numerical increase in total triglycerides content in *longissimus* muscle of NBW pigs (159.21 μmol/g protein vs. 174.94 μmol/g protein), but no significant difference was found. However, they found a significant interaction between dietary resveratrol and birth weight, which indicated that dietary resveratrol reduced the total triglycerides content in the *longissimus* muscle of IUGR pigs, which is vary in NBW pigs and showed that the regulatory effect of dietary resveratrol on fat deposition in muscle vary between NBW pigs and IUGR pigs [109]. It seems that a tissue-specific manner of dietary resveratrol on fat deposition in muscle and adipose tissue of pigs existed. But the underlying molecular mechanisms of dietary resveratrol on fat deposition need further studies.

### Health

#### Intestinal morphology and barriers

Intestinal health is critical for the growth and development of weaned piglets with immature digestive systems [124]. A previous study reported that dietary supplementation of resveratrol increased the villus height, villus height/crypt depth ratio, and the mRNA expression levels of zonula occludens 1 (*ZO*-*1*) and *IL*-*10* and reduced the crypt depth in the jejunum of weaned piglets, and the supplementation concentration at 300 mg/kg was more effective than that at 150 mg/kg [17]. Gan et al. [91] also discovered that resveratrol supplementation reduced IL-1β and TNF-α levels and mRNA expression levels of *IL-1β*, *TNF-α,* and Toll-like receptor 4 (*TLR4*) and increased *IL-10* mRNA expression levels and *Lactobacillus* copies in the jejunum and ileum of weaned piglets. Furthermore, resveratrol supplementation could reduce diamine oxidase (DAO) and *D*-lactate contents, increase the antioxidant activity and mRNA expression levels of antioxidant enzymes *CAT* and *SOD1* and tight junction protein *Occludin* (*OCLN*) and *Claudin 1* (*CLDN1*) in the jejunum and ileum, and this study also showed that methylations of *ZO-1*, heme oxygenase 1, *OCLN,* and *CLDN1* are essential for the resveratrol to have positive effects on intestinal health [88]. For IUGR piglets, resveratrol supplementation increases the villus height and elevates the antioxidant capacity by activating the NRF2 pathway and decreases the intestinal permeability and apoptosis index in the jejunum [92]. Meanwhile, the shift in gut microbiota was also observed when resveratrol supplementation at 300 mg/kg in the diet of IUGR piglets, including enrichment of Bacteroidetes, *Provotella*, *Faecalibaterium* and *Parabacterioides* and reduction in Proteobacteria, *Escherichia* and *Actinobacillus* [92].

The antioxidant potential of resveratrol may be the essential reason for its regulation of intestinal health. The intestinal protective effect of dietary resveratrol was shown in oxidative stress models of piglets. For example, resveratrol supplementation at a 100 mg/kg concentration after diquat treatment for 13 d could decrease the accumulation of MDA, hydrogen peroxide, and ROS and increase the total antioxidant capacity (T-AOC) in the jejunum of weaned piglets [97, 125]. Resveratrol supplementation at a 300 mg/kg concentration for 14 d before diquat treatment improved the jejunal SOD activity and reduced cell apoptosis percentage in the jejunum without alleviating the adverse effects induced by diquat on intestinal morphology and tight junction protein expression, showing that more time might be necessary for resveratrol to show the beneficial effects [41]. Resveratrol supplementation at 10–90 mg/kg concentrations for 14 d before and for 6 d after diquat treatment effectively improved mRNA and protein expression levels of tight junction proteins, alleviated oxidative stress and inflammation by activating NRF2/aryl hydrocarbon receptor pathways, increased villus height and villus height/crypt depth ratio in the jejunum, and reduced DAO and *D*-lactate levels in the plasma of weaned piglets [98, 99]. Therefore, the effectiveness of resveratrol on the intestinal health of piglets suffering oxidative stress caused by diquat might depend on the supplementation phase, and resveratrol supplementation before and after diquat treatment seems to be more effective. Additionally, DON, a toxic metabolite produced by *Fusarium graminearum*, induces severe oxidative stress, which shows toxicity against piglets [126]. Resveratrol supplementation elevated the jejunal mRNA and protein expression levels of ZO-1 and OCLN, increased villus height, villus height/crypt depth ratio, and goblet cells, and reduced serum *D*-lactate and DAO levels when the 21 d weaned piglets were exposed to DON [21]. In contrast, the enhancement of jejunal morphology, mitochondrial potential, and antioxidant enzyme activity with no effect on barrier permeability was observed when DON-exposed piglets weaned on d 28 were treated using resveratrol supplementation at the same concentration. This difference might be from the various physiological conditions of piglets weaned on d 21 and 28 and the various application treatment levels of DON, which should be further investigated. For the inflammatory response caused by DON, resveratrol supplementation in the diets of piglets weaned on d 21 and 28 could decrease the mRNA expression of proinflammatory factors, reducing the adverse effects on intestinal health [21, 102]. Additionally, in the diets of weaned pigs, resveratrol supplementation could alleviate the mitochondria damage in the ileum caused by DON and minimize mitophagy-related gene expression [101].

#### Mitochondrial function

Redox status equilibrium is mediated by ROS production and antioxidant system [127]. The mitochondrial involves the regulation of redox homeostasis, and resveratrol potentially benefits the mitochondrial function [128]. For example, resveratrol supplementation at a concentration of 300 mg/kg diet of early weaning piglets enhanced hepatic mitochondrial electron transport chain complex I activity [87]. Furthermore, previous studies showed that resveratrol supplementation promoted hepatic mitochondrial biogenesis and ATP production in the suckling IUGR piglets, enhancing the energy metabolism [93, 94]. Zhang et al. [95] also reported that resveratrol supplementation in artificial milk of the suckling IUGR piglets improved the hepatic mitochondrial biogenesis by mediating the SIRT1/PGC-1α axis, improved ATP production by upregulating the mRNA expression of ATP synthase alpha subunit and ATP synthase beta polypeptide and improved the activity of complexes III. The improvement of phosphorylation of AMPK and liver kinase B1, mRNA level of acyl-CoA synthetase long-chain family member 1, *CPT*-*1A* and *PPARα* also contributed to mitochondrial fatty acid oxidation stimulated by resveratrol in the liver of the suckling IUGR piglets [95]. In some oxidative stress models, resveratrol supplementation could maintain normal mitochondrial function. For example, the resveratrol treatment could improve the mitochondrial structure and function with a decrease in ROS production and increase in membrane potential, mitochondrial DNA content, and electron transport chain complex I-IV activities by mitophagy improvement in an oxidative stress model of piglets induced by diquat [97]. However, dietary resveratrol could not affect hepatic redox status, mitochondrial biogenesis, and complex activity when the resveratrol was added for 14 d before the oxidative stress treatment [41, 96].

#### Inflammation and immunity

The host immunity system, comprising the innate and adaptive immune systems, is a critical barrier against pathogen invasion [129]. Resveratrol has been proven to exhibit immunoregulation capability. For example, a previous study showed that supplementation of dry suspension containing resveratrol stimulated the proliferation of peripheral blood and splenic lymphocytes, enhanced the host immune responses of classical swine fever and foot-and-mouth disease vaccines, promoted immunoglobulin (Ig)G production, regulated the release of interferon (IFN)-γ, and downregulated the release of TNF-α [90]. Pathogenic bacteria and virus infection can stimulate the cell, activating the immune system and releasing inflammatory signals [130, 131]. Ahmed et al. [14] showed that dietary resveratrol improved the IgG content in weaned piglets challenged with *Salmonella typhimurium* and *E. coli*. In vitro resveratrol alleviated *E. coli* K88 infection-induced damage, including decreased cell viability, reduced tight junctions, mitochondrial dysfunction, and autophagy in the porcine intestinal epithelial cell by activating SIRT1 signaling [132]. For viruses, resveratrol treatment relieved the inflammation response by inhibiting the TNF-α production and inhibited rotavirus infection by enhancing the IFN-γ content and CD4^+^/CD8^+^ ratio, decreasing the diarrhea index [103]. *Pseudorabies virus* infection results in severe disruption of the pig production and massive economic loss [133]. Zhao et al. [104] showed that resveratrol treatment for 7 d before and for 21 d after *Pseudorabies virus* infection could reduce the lesional scores of organs (brain, lung, kidney, liver, spleen and heart), increase the levels of TNF-α, IFN-α, IFN-γ, and IL-12 of the serum in weaned piglets, decreasing the inflammation, viral reproduction and lethality.

### Reproductive performance and maternal regulation

Because of its remarkable antioxidant, anti-inflammatory, and metabolic regulation features, resveratrol has been indicated as a therapeutic agent for pregnancy complications in rats, mice, and Japanese macaque models of complicated pregnancy (maternal dietary manipulations, gestational diabetes and maternal hypoxia) and shows beneficial effects on maternal, embryo and offspring (as reviewed in [134, 135]). Meng et al. [110] reported that dietary resveratrol supplementation in sows’ diets during gestation and lactation increases the antioxidant status of sows and piglets and regulates the antioxidant gene expression and Kelch-like ECH-associated protein 1 (KEAP1)-NRF2 pathway in the placenta. They also discovered that maternal dietary resveratrol increases the weaning weight and litter weaning weight of offspring [110]. Similarly, a recent study also reported that dietary resveratrol increases the litter weight gain at high summer temperature and increases the number of piglets born alive at both high temperature and moderate temperature [113]. The improved effects of dietary resveratrol on the milk were observed, including the increased lactose and fat contents and increased IgA, IgG, and IgM contents [112, 113]. Furthermore, Meng et al. [18] discovered that maternal dietary resveratrol increases the ADG of piglets during weaning and alleviates weaning-associated diarrhea and intestinal inflammation in porcine offspring during weaning and postweaning, possibly owing to the increased proportion of butyrate-producing bacteria in piglets. Zhao et al. [113] also observed that maternal dietary resveratrol increases the abundances of *Lactobacillus* and *Alloprevotella* and decreases the abundance of *Escherichia-shigella* in the faces of piglets under high summer temperature. Additionally, Sun et al. [112] showed that maternal dietary resveratrol improved the enzyme activity and gene expression related to lipolysis, fatty acid uptake from circulating triacylglycerols and lipogenesis in the adipose tissue of weaning piglets, which indicated that the fat metabolism of weaning offspring was improved by resveratrol. In the continuity study, the same batch of weaning piglets was fed until the finish stage. The backfat thickness of finishing pigs was increased by maternal dietary resveratrol supplementation [111]. The beneficial effects of resveratrol have been demonstrated to be linked with epigenetic regulatory regulation [136]. Previous studies found that maternal dietary resveratrol showed beneficial regulatory effects on offspring via epigenetic modification [137]. Thus, the maternal effects of dietary resveratrol in pigs may mediate through epigenetic mechanisms, which need to be further studied in the future.

## Application of resveratrol in poultry production

In poultry production, the effects of dietary resveratrol have been studied in quails, ducks, broilers, and hens, as indicated in Table 2. In this section, the effects of dietary resveratrol on the growth performance, meat and egg quality and health state of poultry will be reviewed.

**Table 2:** Application of resveratrol in poultry production

Animal	Dosage	Stress model/ study design	Main beneficial effects	Reference
Quails 5-week-old	200, 400 mg	12-week feeding	Exhibited a linear decrease in serum and egg yolk MDA and a linear increase in serum vitamin E	[15]
Laying hens 60-week-old	0.5, 1.0, 2.0, and 4.0 g/kg	8-week feeding	Reduced FCR; improved Haugh unit and albumen height of eggs and decreased contents cholesterol in the yolk	[138]
Laying hens 210-day-old	200, 400, and 800 mg/kg	1-month feeding	Increased egg production rate and ADFI and decreased feed-to-egg ratio; reduced egg cholesterol content; extended egg shelf life and improved egg sensory scores and yolk index	[139]
Ducks 1-day-old	150, 300, and 450 mg/kg	21-d feeding	Improved meat quality in leg muscle, including meat color, pH, drip loss and shear force	[140]
Broilers 21-day-old	400 mg/kg	21-d feeding	Decreased L*, pH decline, drip loss, and lactate content in pectoralis major muscle; increased antioxidant state and PGC-1α and nuclear NRF1 mRNA expression and citrate synthase activity	[141]
Broilers 21-day-old	400 mg/kg	Transport stress, 21-d feeding	Improved feed conversion ratio and tended to improve final BW; increased glycogen content, LDH activity, and drip loss in the muscle of transported broilers	[142]
Broilers 1-day-old	300, 600 mg/kg	*E. coli,* 42-d feeding	Increased ADG and decreased FCR of broilers challenged with *E. coli*; increased villus length to crypt depth and decreased *E. coli* number in challenged chickens	[143]
Ducks 1-day-old	500 mg/kg	Aflatoxin B1, 42-d feeding	Increase II-phase enzyme activity, activate NRF2 signal pathway, and protect oxidative stress and inflammatory reaction in the liver induced by AFB1	[144]
Ducks 1-day-old	500 mg/kg	Aflatoxin B1, 70-d feeding	Alleviated ileum injury induced by AFB1, decreased the production of AFB1-DNA adducts and reduced DNA damage and oxidative stress via NRF2/KEAP1 and NF-κB/NLRP3 signaling pathways	[145]
Chicken 1-day-old	200, 400 and 800 mg/kg	Conventional vaccinations, 40-d feeding	Increased ADG and antibody titers against Newcastle disease virus and avian influenza viruses H5 and H9	[146]
Ducks 1-day-old	400 mg/kg	LPS treatment, 28-d feeding	Improved final BW and ADG before LPS treatment; alleviated LPS-induced inflammatory response in liver and intestine and increased tight junction protein expression	[147, 148]
Ducks 1-day-old	300, 400 and 500 mg/kg	70-d feeding	Increased pH and reduced MDA, carbonyl contents, and shear force, thereby improving water mobility and distribution, drip loss and cooking loss of duck meat; inhibited the formation of carbonyl and dityrosine and reduced the loss of sulfhydryl in frozen-thawed duck breast meat	[149, 150]
Quails 55-day-old	200, 400 mg/kg	Heat stress, 12-week feeding	Increased ADFI and egg production; increased hepatic SOD, CAT, and GPX activities as well as NRF2 expression, decreased hepatic MDA concentrations and Hsp70, Hsp90, and NF-κB expressions	[151]
Black-boned chickens 42-day-old	200, 400 and 600 mg/kg	Heat stress, 15-d feeding	Increased feed intake and BW gain; improved villus morphology, numbers of goblet cells and lymphocytes and regulated heat shock protein expression in the thymus and intestine	[19, 152]
Broilers 21-day-old	400 mg/kg	Heat stress, 21-d feeding	Increased final BW of heat-stressed broilers; decreased crypt depth and *E. coli* populations, and increased villus height, goblet cells numbers, populations of *Lactobacillus* and *Bifidobacterium*; Increased a*, pH_24h_, and decreased L*, drip loss and muscle MDA content of heat-stress broilers	[20, 153]
Broilers 28-day-old	200, 350, and 500 mg/kg	Heat stress, 14-d feeding	Improved ADG and decreased rectal temperature of heat-stressed broilers; lowered contents of corticosterone and adrenocorticotropic hormone	[154]
Broilers 28-day-old	500 mg/kg	Heat stress, 14-d feeding	Increased ADFI and ADG under heat stress; decreased crypt depth, increased villus height; increased gene expression related to gut barrier and decreased gene expression related to inflammation; improved immune organ index and inhibited the NF-κB, MAPK, and PI3K/AKT signaling pathway in spleen	[66, 155]
Broilers 21-day-old	400 mg/kg	Heat stress, 21-d feeding	Increased BW and ADG under heat stress; increased relative jejunum weight, relative length of ileum, jejunal villus height, activities of GPX and GST, and mRNA levels of *NRF2* and *SOD1* under heat stress	[156]
Ducks 60-day-old	400 mg/kg	Heat stress, 15-d feeding	Increased the ratio of villus height to crypt depth and the number of goblet cells and reduced the histopathological damage of jejunum; activated SIRT1-NRF1/NRF2 signaling pathways, improved ATP level of jejunum	[157]
Broilers 28-day-old	500 mg/kg	Heat stress, 14-d feeding	Decreased FCR under heat stress; decreased levels of corticosterone and adrenocorticotropic hormone caused and reduced heat stress-induced apoptotic cells and apoptosis genes	[158]

*MDA* malondialdehyde, *PGC-1α* peroxisome proliferators-activated receptor gamma coactivator-1 alpha, *ADFI* average daily feed intake, *ADG* average daily gain, *FCR* feed conversion ratio, *BW* body weight, *NRF1* nuclear respiratory factor 1, *CAT* catalase, *GPX* glutathion peroxidase, *NRF2* nuclear factor erythroid 2-related factor 2, *NF-κB* nuclear factor κB, *KEAP1* kelch-like ECH-associated protein 1, *SOD* superoxide dismutase, *MAPK* mitogen-activated protein kinases, *LPS* lipopolysaccharides, *GST* glutathione S-transferase, *SIRT1* sirtuin 1, *LDH* lactate dehydrogenase, *AFB1* aflatoxin B1, *Hsp* heat-shock protein 70, *PI3K* phosphoinositide-3 kinases, *AKT* protein kinase B, *NLRP3* NOD-like receptor protein 3, a* redness, L* lightness

### Growth performance

In poultry, accumulating studies have explored the impacts of dietary resveratrol supplementation on the growth performance of broilers, ducks, and quails. In broilers, Zhang et al. [142] discovered that supplementation of 400 mg/kg resveratrol increased the final BW and reduced the feed conversion ratios (FCR) of broilers. Feng et al. [138] explored the influence of various levels of dietary resveratrol (0, 0.5, 1.0, 2.0, and 4.0 g/kg) on the laying hens, discovering that dietary 2.0 g/kg of resveratrol decreased the FCR. In ducks, Yang et al. [147] reported that dietary 400 mg/kg resveratrol increased the final BW and ADG after 28 days of feeding. Additionally, dietary resveratrol exhibits obvious improved effects on the growth performance of poultry in some adverse factor challenges and stress models. Mohebodini et al. [143] studied the effects of dietary resveratrol supplementation in broiler chickens challenged with *E. coli* and discovered that dietary resveratrol increased BW gain and reduced the FCR of broiler chickens. They also discovered that dietary 600 mg/kg resveratrol has a similar effect to colistin sulfate, and both resulted in similar growth performance to that of the unchallenged broiler chickens. The previous study also explored the effects of dietary resveratrol (0, 200, 400, and 800 mg/kg) on chickens who received conventional vaccinations and discovered that the ADG of chickens quadratically increased with increasing resveratrol supplementation [146]. Heat stress is considered the most significant challenge in poultry production worldwide, always causing increased mortality and reduced growth performance. In poultry, supplementation of resveratrol in diets increases the feed intake [19, 66, 151], ADG [19, 66, 154, 156], and BW [20, 156] and minimizes FCR [158], which shows that dietary resveratrol could effectively alleviate growth inhibition caused by heat stress.

### Meat quality and egg quality

Similar to pigs, dietary resveratrol increases the meat quality of poultry. In Pekin ducks, Yu et al. [140] studied the influence of four levels of resveratrol (0, 150, 300, and 450 mg/kg) on the meat quality and reported that dietary resveratrol increased the a*_24h_ and b*_24h_ of breast muscle and a*_45min_ of leg muscle, also reduced shear force, and L*_45min_ of breast muscle and drip loss, shear force, and L*_45min_ of leg muscle. Similarly, Jin et al. [149] also note that dietary resveratrol improves the meat quality of ducks, as demonstrated by increased post-slaughter pH, and decreased shear force, drip loss, and cooking loss, which may be because of the enhanced antioxidant capacity and inhibited lipid and protein oxidation. In broilers, dietary resveratrol decreased L*_45min_, pH decline, drip loss, and MDA content, and increased the T-AOC and CAT activity in the pectoralis major muscle [141]. Moreover, increased *PGC-1α* and nuclear respiratory factor 1 (*NRF1*) mRNA expression and citrate synthase activity were found in the broilers’ muscle [141]. Moreover, dietary resveratrol improves the meat quality of broilers under heat stress. Zhang et al. [153] reported that dietary resveratrol supplementation in broilers alleviated heat stress induced reduction in meat quality, increased the a*, pH_24h_, T-AOC and CAT activities, and reduced the L*_24h_, drip loss, and MDA content in muscle.

Additionally, the egg quality was observed to be regulated by dietary resveratrol. Sahin et al. [15] discovered that egg yolk MDA concentration reduced linearly in response to increasing dietary resveratrol level, which may prolong shelf life and benefit consumers; Moreover, the Haugh unit tended to be increased, and the diameter and width of yolk were linearly reduced by dietary resveratrol supplementation [15]. Feng et al. [138] also found that dietary 2.0 g/kg of resveratrol improved the egg quality with increased Haugh unit and albumen height and reduced egg yolk cholesterol. Zhang et al. [139] discovered that 200 mg/kg resveratrol increased egg yield and reduced the feed to egg ratio of laying hens, whereas 400 mg/kg dose was associated with better lipid metabolism (reduced serum cholesterol and triglycerides), decreased egg cholesterol content, extended egg shelf life and improved yolk index and egg sensory scores. Haugh unit and albumen height are important indicators for evaluating freshness and quality of eggs [159]. These results supported that dietary resveratrol supplementation could be beneficial to laying hens for extending the shelf life and reducing the cholesterol of the eggs.

### Health

#### Immune function

Commercial poultry is vaccinated routinely to protect the birds against infection or disease caused by many pathogens to decrease the mortality rate and infection susceptibility. In chickens under normal conventional vaccinations, dietary resveratrol decreases inflammation-related genes in the liver and spleen and increase CD4^+^ cell and CD4^+^/CD8^+^ ratio in peripheral blood and IgM content in serum [146]. Lipopolysaccharides (LPS) are endotoxins, hazardous and toxic inflammatory stimulators released from the outer membrane of Gram-negative bacteria. In poultry, dietary resveratrol was shown to alleviate the inflammatory reactions induced by LPS in vitro and in vivo. Chicken peripheral blood lymphocytes treated with resveratrol showed reduction in the activity of NF-κB, levels of TNF-α and ROS and apoptosis-related protein expression, increased the viability of lymphocytes and reduced the apoptotic rate after continuous stimulation by LPS [160]. In ducks, a recent study reported that dietary resveratrol alleviated LPS-induced inflammatory response with a decrease in the levels of IL-1β and IL-6 in the plasma and liver through TLR4/NF-κB signaling pathways [147]. Yang et al. [148] also discovered that dietary resveratrol could alleviate LPS-induced intestinal dysfunction and increase mRNA levels of *CLDN1, OCLN, ZO-1* and protein expression of CLDN1, which may be related to the regulation of TLR4/NF-κB signaling pathway and its downstream genes.

#### Health-related to heat stress

Heat stress has strong adverse effects on the growth performance, intestinal morphology, mortality, and welfare of broilers, which can be alleviated by nutrition regulation. Because of its antioxidant and anti-inflammatory qualities, dietary resveratrol alleviates heat stress in quails [151], chickens [19, 152], broilers [20, 66, 153–156, 158], and ducks [157]. Previous studies discovered that dietary resveratrol increased SOD, CAT, and GPX enzyme activity and GSH content and reduced MDA levels in the liver and serum in heat-stressed chickens [19, 151]. Additionally, hepatic NRF2 protein expression in heat-stressed quails was increased, and heat shock protein (Hsp)70, Hsp90, and NF-κB protein expressions were reduced with increasing resveratrol supplementation levels [151]. In chickens, heat stress-induced overexpression of *Hsp27*, *Hsp70*, and *Hsp90* mRNA in the bursa of Fabritius and spleen was attenuated, and low expression of *Hsp27* and *Hsp90* mRNA in the thymus upon heat stress was increased by dietary resveratrol [19]. In the spleen of broilers, dietary resveratrol reduced heat stress-induced apoptotic cells number and mRNA expression levels of genes involved in apoptosis induced by heat stress, including B-cell lymphoma-2, apoptotic protease activating factor-1, and murine double minute 2 [158]. He et al. [154] and Meng et al. [158] observed that dietary resveratrol reduced rectal temperature and lowered the contents of corticosterone and adrenocorticotropic hormone in heat-stressed broilers, showing that the stress state was alleviated by dietary resveratrol.

Additionally, heat stress usually causes impaired intestinal health in poultry. Recent research found that dietary resveratrol supplementation shows beneficial improvements in the intestinal health of heat-stressed poultry, as shown in intestinal morphology, oxidative stress, and inflammatory state, and intestinal barrier function. In intestinal morphology, studies have reported that dietary resveratrol increased the numbers of goblet cells and lymphocytes, villus height, and villus height/crypt depth ratio and decreased the crypt depth and histopathological damage in the intestine of heat-stressed poultry [20, 152, 157]. The increased mRNA expression of mucin glycoproteins 2, secreted immunoglobulin, *OCLN*, and *CLDN1* in the intestine of heat-stressed broilers was also observed [66], indicating that gut barrier function was improved by dietary resveratrol. The heat shock protein expression in the gut of heat-stressed poultry was also regulated by dietary resveratrol, including Hsp60, Hsp70, and Hsp90 [66, 152, 157]. The antioxidant and anti-inflammatory qualities of resveratrol mainly contribute to the beneficial roles of heat stress, which has been shown by the regulation of related pathways and genes. Yang et al. [157] found that dietary resveratrol activated the SIRT1-NRF1/NRF2 signaling pathway and improved ATP level. This may contribute to the increased SOD and CAT antioxidant enzyme activities in the jejunum of ducks exposed to acute heat stress. Wang et al. [156] observed that the activities of GPX and glutathione S-transferase and mRNA levels of *NRF2* and *SOD1* were increased, and *KEAP1* mRNA levels were reduced in the jejunum of broilers under heat stress. Alternatively, dietary resveratrol could attenuate heat stress-impaired intestinal microbiota balance, as indicated by reduced the population of *E. coli* and increased the population of *Lactobacillus* and *Bifidobacterium* in the intestine [20]. The beneficial role of dietary resveratrol was also observed in the laying performance of heat-stressed poultry. In the heat-stressed quail, Sahin et al. found that egg production, hepatic SOD, CAT, and GPX activities and NRF2 expression were linearly increased while hepatic MDA concentrations and Hsp70, Hsp90, and NF-κB expressions were linearly reduced with increasing supplemental resveratrol levels [151].

#### Response to mycotoxin

Mycotoxins are secondary metabolites of different species of fungi that could cause chronic or acute toxicity in animals [161]. In ducks, supplementation with 500 mg/kg resveratrol significantly ameliorates aflatoxin B1-induced oxidative stress, inflammation, mitochondrial dysfunction, and DNA damage in the ileum, which may be related to the reduced mRNA expression of cytochrome P4501A1 and cytochrome P4501A2 and NRF2/KEAP1 and NF-κB/NOD-like receptor protein 3 (NLRP3) signaling pathway [145]. Liu et al. [144] also indicated that dietary resveratrol in ducks inhibited acute liver injury induced by aflatoxin B1, regulating phase-II metabolism enzymes, NRF2, SIRT1, NF-κB, and cell apoptosis pathway.

## Application of resveratrol in ruminant production

Few studies have investigated the effects of dietary resveratrol on ruminants, as summarized in Table 3. In ruminants, previous studies have failed to observe the improved effects of dietary resveratrol on the growth performance of calves [162]. In sheep, dietary resveratrol was shown to enhance the nutrient digestibility of dry matter, organic matter, neutral detergent fiber, acid detergent fiber, and nitrogen [164]. In fattening goats, a recent study indicated that dietary 150 mg/kg resveratrol increased ADG, final weight, and hot carcass weight, while dietary 600 mg/kg resveratrol exhibited the opposite effect, as evidenced by lower ADFI and ADG and higher F/G [165]. They also found that dietary resveratrol improved the impact on meat quality, as evidenced by increased intramuscular fat content and redness and reduced shear force in muscle [165]. Additionally, a previous study reported that dietary resveratrol reduced CH_4_ output scaled in sheep [16]. Dietary resveratrol was also shown to regulate the rumen microbiota composition. Zhang et al. [162] discovered that feeding 4 mg/kg BW resveratrol increased the population of *Desulfovibrio* and reduced the methanogenic archaea population in Holstein’s calves for a short period. Ma et al. [16] discovered that 0.25 g/d resveratrol increased the ruminal populations of *Fibrobacter succinogenes*, *Ruminococcus albus,* and *Butyrivibrio fibrisolve*ns and reduced protozoa and methanogens in the rumen of sheep. Because of the anti-inflammatory features, resveratrol was also shown to inhibit inflammation in sheep. The study by Liang et al. [166] reported that injected intravenously with resveratrol attenuated the LPS-evoked inflammatory responses in lambs by inhibiting expression levels of inflammatory cytokines and blocking NF-κB and MAPK signaling pathways. However, the effects of dietary supplementation and injection vary. Thus, dietary resveratrol’s effect on the inflammatory state of ruminants needs further research in the future.

**Table 3:** Application of resveratrol in ruminant production

Animal	Dosage	Stress model/ study design	Beneficial effects	Reference
Calves 7-day-old	4 mg/kg BW	173-day-feeding	Increased *Desulfovibrio* population; decreased methanogenic archaea population and pH in remen	[162]
Sheep 60 kg	0.125, 0.25, 0.5, 1.0, and 2.0 g/d	8-day-feeding	Reduced CH_4_ and CO_2_ emission in sheep	[163]
Sheep 60 kg	0.25 g/d	16-day-feeding	Reduced CH_4_ and CO_2_ output scaled; improved apparent digestibility of DM, OM, NDF, ADF, nitrogen, and ME; reduced energy losses in CH_4_ output	[164]
Ewes 12-month-old	0.25 g/d	29-day-feeding	Improved the apparent digestibility of OM, N, NDF, and ADF	[16]
Ewes 18-month-old	0.25 g/d	42-day-feeding	Increased ruminal population of *Fibrobacter succinogenes*, *Ruminococcus albus,* and *Butyrivibrio fibrisolvens*; decreased protozoa and methanogens; decreased CO_2_ and CH_4_ output scaled	[16]
Fattening goats 28.25 kg	0, 150, 300 and 600 mg/kg	120-day-feeding	150 mg/kg resveratrol increased ADG, final BW, and hot carcass weight, while 600 mg/kg resveratrol exhibited opposite effect; increased intramuscular fat content and redness and reduced shear force in the muscle	[165]

*DM* dry matter, *OM* organic matter, *NDF* neutral detergent fiber, *ADF* acid detergent fiber, *ME* metabolic energy, *N* nitrogen, *ADG* average daily gain, *BW* body weight

## Conclusions and perspectives

**Fig. 4:**
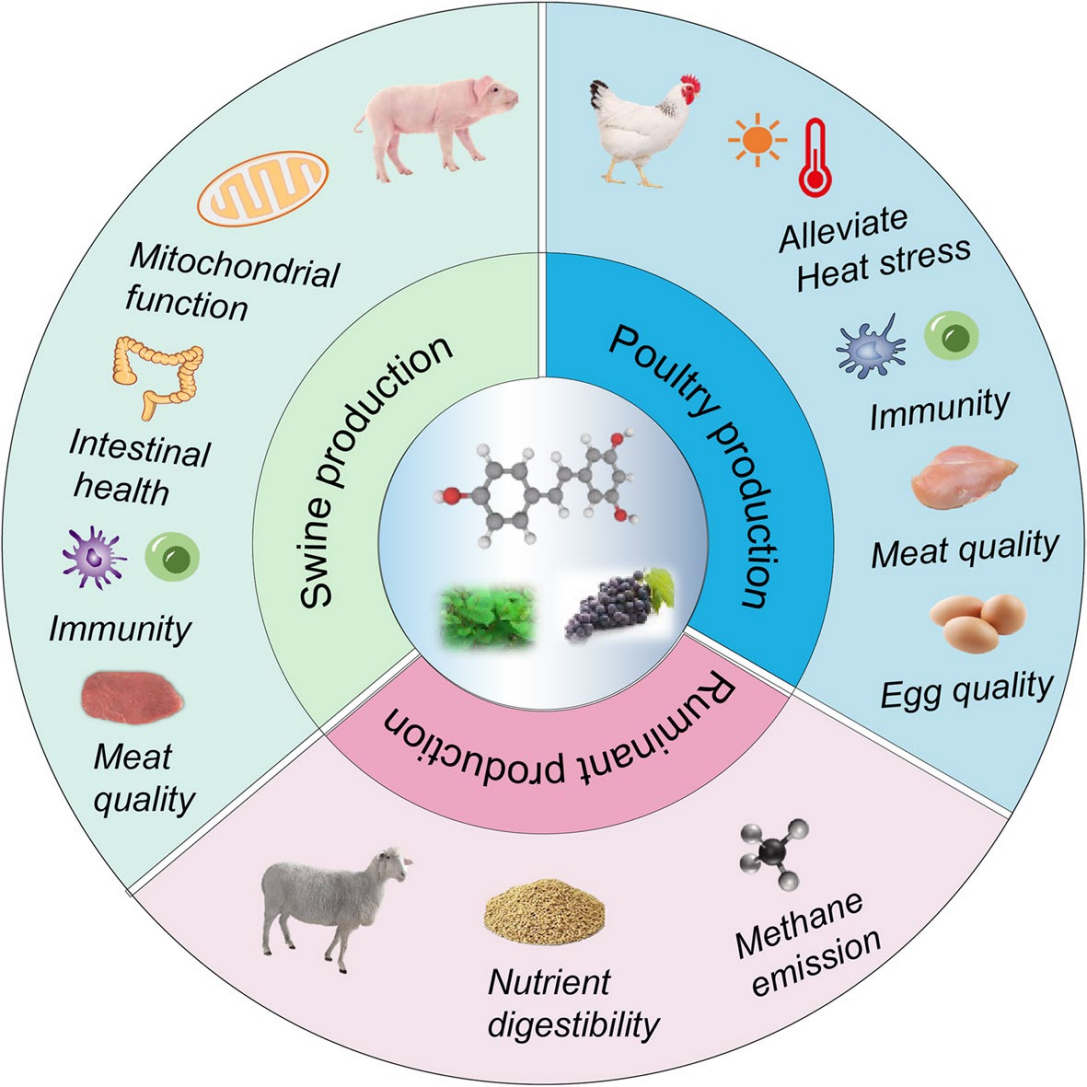
The summary of beneficial effects of dietary resveratrol in animal production

This review summarized and reviewed the research on resveratrol in pigs, poultry, and ruminant. The research so far suggested that owing to the antioxidant, anti-inflammatory and metabolic regulation qualities, dietary resveratrol improves the health state and growth performance of animals under adverse or stress states, especially in poultry, and shows beneficial effects on the reproductive performance of sows. Dietary resveratrol could enhance the meat and fat quality in pigs, poultry, and ruminants and show beneficial roles in egg quality. In ruminants, dietary resveratrol could reduce methane emissions, which is conducive to environmental protection (as summarized in Fig. 4). However, the specific supplementation dose in various animals and mechanism of action, especially the gut microbiota interaction behind the effects of resveratrol, should be further studied in the future. Additionally, more studies with many animals are required to study its impact on the growth performance and reproductive performance of animals. In the background of feed antibiotics prohibition, resveratrol, as a plant functional substance, deserves further research and application in animal production. We hoped that this review could provide theoretical basis and research ideas for the research and application of resveratrol.

## Data Availability

Not applicable.

## Abbreviations

a*: Redness
ACC: Acetyl-CoA carboxylase
ADFI: Average daily feed intake
ADG: Average daily gain
AMPK: AMP-activated protein kinase
ATGL: Adipose triglyceride lipase
b*: Yellowness
BW: Body weight
CAT: Catalase
CD4^+^/CD8^+^: Cluster of differentiation 4/cluster of differentiation 8
CLDN1: Claudin 1
COX: Cyclooxygenase
CPT-1: Carnitine palmitoyl transferase-1
DAO: Diamine oxidase
DON: Deoxynivalenol
FAS: Fatty acid synthase
FCR: Feed conversion ratios
F/G: Feed to gain ratio
GPX: Glutathione peroxidase
GSH: Reduced glutathione
HSL: Hormone sensitive lipase
Hsp70: Heat shock protein (Hsp)70
IFN-γ: Interferon (IFN)-γ
IgG: Immunoglobulin (Ig)G
IL-1β: Interleukin (IL)-1β
IUGR: Intrauterine growth retardation
KEAP1: Kelch-like ECH-associated protein 1
L*: Lightness
LPL: Lipoprotein lipase
LPS: Lipopolysaccharides
MAPK: Mitogen-activated protein kinases
MDA: Malondialdehyde
MRP: Multidrug resistance protein
MyHC: Myosin heavy chain
NBW: Normal birth weight
NF-κB: Nuclear factor kappa B
NLRP3: NOD-like receptor protein 3
NRF1: Nuclear respiratory factor 1
NRF2: Nuclear factor-erythroid 2-related factor 2
OCLN: Occludin
PGC-1α: Peroxisome proliferators-activated receptor gamma coactivator-1 alpha
PPARγ: Peroxisome proliferator activated receptor (PPAR)γ
ROS: Reactive oxygen species
SIRT1: Sirtuin 1
SOD: Superoxide dismutase
T-AOC: Total antioxidant capacity
TLR4: Toll-like receptor 4
TNF-α: Tumor necrosis factor-alpha
ZO-1: Zonula occludens 1
